# Molecular characterization and distribution of *Cryptosporidium* spp., *Giardia duodenalis*, and *Enterocytozoon bieneusi* from yaks in Tibet, China

**DOI:** 10.1186/s12917-019-2172-6

**Published:** 2019-11-21

**Authors:** Yayun Wu, Yankai Chang, Xiangqian Zhang, Yuancai Chen, Dongfang Li, Lu Wang, Shuangjian Zheng, Rongjun Wang, Sumei Zhang, Fuchun Jian, Changshen Ning, Jiakui Li, Longxian Zhang

**Affiliations:** 1 College of Animal Science and Veterinary Medicine, Henan Agricultural University, Zhengzhou, 450002 Henan Province China; 2 International Joint Research Laboratory for Zoonotic Diseases of Henan, Zhengzhou, 450002 Henan Province China; 3 College of Veterinary Medicine, Huazhong Agricultural University, Wuhan, 430070 Hubei Province China; 4 Laboratory of Detection and Monitoring of Highland Animal Disease, Tibet Agriculture and Animal Husbandry College, Linzhi, 860000 Tibet China

**Keywords:** *Cryptosporidium* spp., *Giardia duodenalis*, *Enterocytozoon bieneusi*, Zoonotic, Grazing, Tibet, Yaks

## Abstract

**Background:**

With worldwide distribution and importance for veterinary medicine, *Cryptosporidium* spp., *Giardia duodenalis*, and *Enterocytozoon bieneusi* have been found in a wide variety of vertebrate hosts. At present, few available molecular data can be used to understand the features of genetic diversity of these pathogens in areas without or less intensive farming. Dominated by grazing, Tibet is a separate geographic unit in China and yaks are in frequent contact with local herdsmen and necessary for their daily life. Therefore, to investigate the distribution of these pathogens in yaks of Tibet, 577 fecal specimens were screened using nested PCR for the presence and genotypes of the three intestinal pathogens.

**Results:**

The overall prevalence of *Cryptosporidium* spp., *G*. *duodenalis*, and *E*. *bieneusi* were 1.4% (8/577), 1.7% (10/577), and 5.0% (29/577), respectively. *Cryptosporidium andersoni* (*n* = 7) and *Cryptosporidium bovis* (*n* = 1) were detected by sequence analysis of the SSU rRNA gene. Genotyping at the SSU rRNA and triosephosphate isomerase genes suggested that all *G. duodenalis* positive specimens belonged to assemblage E. Sequence analysis of the internal transcribed spacer gene identified six known *E*. *bieneusi* genotypes: BEB4 (*n* = 11), I (*n* = 6), D (*n* = 5), J (*n* = 2), CHC8 (*n* = 1), and BEB6 (*n* = 1). One subtype (A5,A4,A2,A1) for *C. andersoni* and three multilocus genotypes for *E*. *bieneusi* were identified by multilocus sequence typing.

**Conclusions:**

We report for the first time the status of three enteric pathogens infection simultaneously for grazing yaks in Tibet. Yaks in our study are likely to impose a low zoonotic risk for humans. The molecular epidemiology data add to our knowledge of the characteristics of distribution and transmission for these pathogens in Tibet and their zoonotic potential and public health significance.

## Background

As the commonly considered causes of human cryptosporidiosis, giardiosis, and microsporidiosis, *Cryptosporidium* spp., *Giardia duodenalis*, and *Enterocytozoon bieneusi* have been found in a wide variety of vertebrate hosts. These pathogens can present several typical clinical symptoms, varying from asymptomatic infection to acute or chronic diarrhea. Human infections are acquired by several transmission routes, such as direct contact with infected persons (anthroponotic transmission) or animals (zoonotic transmission) or ingestion of contaminated water or food [1–4]. Additionally, livestock have been identified as a source of some outbreaks of human cryptosporidiosis, giardiosis, and microsporidiosis [5–7]. The One Health concept recognizes that the health of people is connected to the health of animals and the environment and emphasizes the importance to study the distribution and transmission pathways of pathogens (http://www.cdc.gov/onehealth/).

Bovines have been identified as the mammals where *Cryptosporidium* infection is most universally found, and bovine cryptosporidiosis is predominantly attributed to *Cryptosporidium parvum*, *C. bovis*, *C. andersoni* and *Cryptosporidium ryanae* [8, 9]. From the limited studies on yaks, at least nine *Cryptosporidium* species and two genotypes have been described in yaks, including *C. parvum*, *Cryptosporidium hominis*, *C. bovis*, *C. andersoni*, *C*. *ryanae*, *Cryptosporidium ubiquitum*, *Cryptosporidium xiaoi*, *Cryptosporidium struthionis*, *Cryptosporidium canis*, *Cryptosporidium occultus*, *C*. *ryanae* cattle type, and *C*. *ryanae* buffalo type [8–10]. Currently, in terms of genetic variation, molecular biological analyses have shown that *G*. *duodenalis* has at least eight genotypes or assemblages (A-H), with A and B mainly infecting humans and animals and C to H being specifically restricted to nonhuman host species [3, 11]. Assemblage E is the most commonly reported in yaks, followed by assemblages A and B [12]. *E*. *bieneusi* has been genotyped into at least nine phylogenetic groups; zoonotic group 1 (also called the human-pathogenic group) includes almost all *E*. *bieneusi* genotypes from humans and some from animals, whereas groups 2–9 are generally considered host-adapted groups, suggesting no major public health significance [13, 14]. Among 13 genotypes detected in yaks from the three studies available, genotypes BEB4 and J were reported to be the dominant *E*. *bieneusi* genotypes in yaks, which were clustered into group 2. Additionally, genotypes CHN11, CHN12, CHN14, and WCY1 belonged to group 1 and the remainder belonged to group 2 [15–17].

Unlike that in other provinces and municipalities of China, the breeding industry is still dominated by grazing in Tibet, and free-range yaks and other abundant livestock are raised mostly on natural pastures and mountains. In addition to less Concentrated Animal Feeding Operation (CAFO), Tibet is a separate geographic unit with the highest average elevation on earth. Approximately 90% of the world’s yak population is distributed on the Qinghai-Tibet Plateau of China, with 4.9 million in Qinghai, 3.9 million in Tibet, 3.1 million in Sichuan, 0.88 million in Gansu, 0.17 million in Xinjiang, and 0.05 million in Yunnan [18]. As the representative livestock of Tibet and known as the “boat of the plateau”, yaks are in frequent contact with local herdsmen and necessary for their daily life. Fresh fecal material from yaks is dried for cooking fuel, which increases the possibility of herder infection by *Cryptosporidium*, *G*. *duodenalis*, and *E*. *bieneusi*. Prior to our work, there was only an epidemiological study about *Cryptosporidium* and *G*. *duodenalis* in yaks from Damxung County in Tibet, in which no *G*. *duodenalis* infection was found [19]. To address the knowledge gap for the frequency of three enteric pathogens in the region, we conducted a series of experiments aimed at characterizing features of genetic diversity of these pathogens in Tibet. Molecular epidemiology data generated from the results could help in assessment of their zoonotic potential and public health significance in areas without or less intensive farming.

## Results

### *Cryptosporidium*

Among 577 samples (62 from Mainling, 125 from Gongbo’gyamda, 262 from Bayi District, 56 from Gyaca, and 72 from Xaitongmoin), *Cryptosporidium* spp. were detected in eight (1.4%, 8/577) specimens based on the SSU rRNA gene. Infection rates ranged from 0 to 6.9% among the five counties (Table 1), and differences among counties were statistically significant (*P* < 0.05). The highest infection rate (6.9%) was observed in Xaitongmoin, and no *Cryptosporidium* was detected in Mainling or Gyaca. The molecular analysis of eight successfully sequenced *Cryptosporidium*-positive products revealed the presence of two species, *C. andersoni* (*n* = 7) and *C. bovis* (*n* = 1), with *C. andersoni* being predominant (7/8, 87.5%). All *C. andersoni* samples were detected in Bayi District and Xaitongmoin and their sequences showed 100% homology to FJ463172 derived from dairy calves in China. *C. bovis* was only identified in Gongbo’gyamda, with no nucleotide difference compared with KT922231 derived from calves in Ethiopia.

**Table 1:** Species, assemblage, and genotype distribution of three enteric pathogens in the present study

Location	Sample size	Prevalence (%) (95% CI)			Species/Assemblage/Genotype (no.)		
		*Cryptosporidium*	*G. duodenalis*	*E. bieneusi*	*Cryptosporidium*	*G. duodenalis*	*E. bieneusi*
Mainling	62	0	0	12.9 (4.3–21.5)	0	0	BEB4 (8)
Gongbo’gyamda	125	0.8 (0.0–2.4)	2.4 (0.0–5.1)	1.6 (0.0–3.8)	*C. bovis* (1)	E (3)	I (2)
Bayi District	262	0.8 (0.0–1.8)	1.5 (0.0–3.0)	4.6 (2.0–7.1)	*C. andersoni* (2)	E (4)	BEB4 (6), I (4), J (2)
Gyaca	56	0	3.6 (0.0–8.6)	5.4 (0.0–11.4)	0	E (2)	CHC8 (1), D (2)
Xaitongmoin	72	6.9 (0.9–13.0)	1.4 (0.0–4.2)	5.6 (0.1–11.0)	*C*. *andersoni* (5)	E (1)	D (3), BEB6 (1)
Total	577	1.4 (0.4–2.3)	1.7 (0.7–2.8)	5.0 (3.2–6.8)	*C*. *andersoni* (7) *C*. *bovis* (1)	E (10)	BEB4 (14), I (6), D (5), J (2), CHC8 (1), BEB6 (1)

The *C. andersoni*-positive samples were characterized by MLST, and four of seven samples were simultaneously amplified based on four microsatellite/minisatellite loci (MS1, MS2, MS3, and MS16). One MLST subtype (A5,A4,A2,A1) was found in the present study.

### *Giardia duodenalis*

For *G. duodenalis*, ten samples (1.7%, 10/577) were tested positive using the SSU rRNA and *tpi* genes. *G. duodenalis* was observed in all counties but Mainling (Table 1), and the infection rates of *G. duodenalis* were 3.6% (Gyaca), 2.4% (Gongbo’gyamda), 1.5% (Bayi District), and 1.4% (Xaitongmoin); rates did not differ significantly by region (*P* > 0.05). Analysis of eight SSU rRNA sequences and six *tpi* sequences showed that all sequences belonged to *G*. *duodenalis* assemblage E. At the SSU rRNA locus, all the assemblage E sequences obtained here (*n* = 8) showed 100% homology to KX259145 previously recognized from sika deer in China. By comparison with *G. duodenalis tpi* sequences available on GenBank, the sequences (*n* = 6) were all identical to isolate KY710747 derived from dairy cattle in China.

### Enterocytozoon bieneusi

**Fig. 1:**
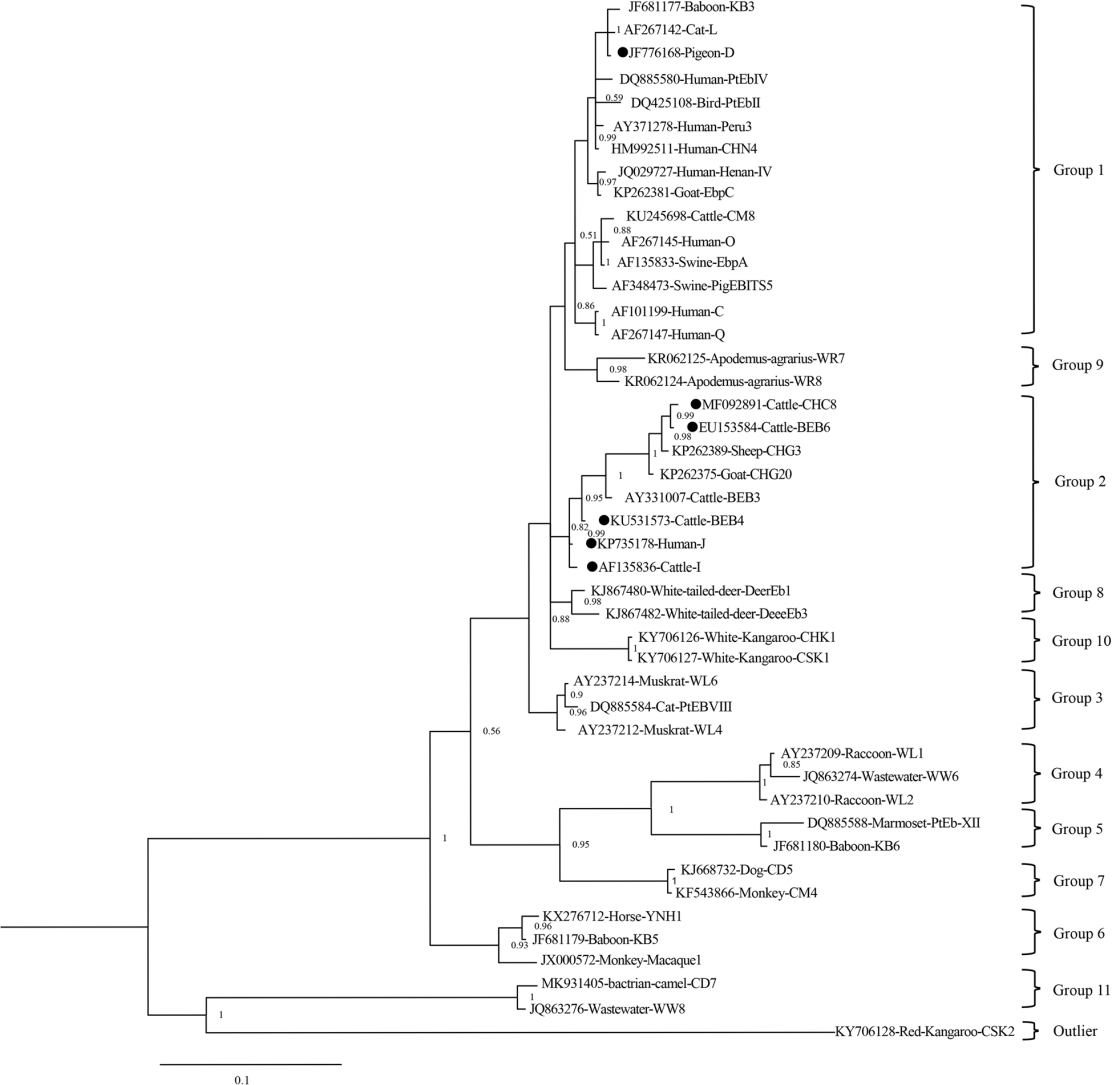
Phylogenetic tree based on Bayesian analysis of the *E*. *bieneusi* ITS genotypes. Statistically significant posterior probabilities are indicated on the branches. Each sequence was labeled with its accession number, host origin, and genotype designation. CSK2 (isolated from a kangaroo in China with GenBank accession no. KY706128) was considered as outlier. Genotypes detected in this study are indicated by filled circles

Among the 577 yaks examined, 29 (5.0%, 29/577) were tested positive for *E*. *bieneusi* based on the ITS region. *E*. *bieneusi* was seen in all counties, with infection rates ranging from 1.6 to 12.9% (Table 1). Yaks in Mainling had the highest infection rate (12.9%) whereas those in Gongbo’gyamda had the lowest (1.6%) occurrence, and infection rates differed significantly between five counties (*P* < 0.05). All positive samples were successfully sequenced, and six previously described genotypes were identified: BEB4 (*n* = 14), I (*n* = 6), D (*n* = 5), J (*n* = 2), CHC8 (*n* = 1), and BEB6 (*n* = 1). The evolutionary relationships and zoonotic potential were analyzed by Bayesian analysis of the *E*. *bieneusi* ITS genotypes. Genotype D was clustered into group 1 (high zoonotic potential) in the phylogenetic analysis. Genotypes I, J, BEB4, CHC8, and BEB6 were clustered into group 2 (Fig. 1). The dominant genotype BEB4 was detected in Mainling and Bayi District; genotype I was observed in Gongbo’gyamda and Bayi District; genotype D was seen in Gyaca and Xaitongmoin; and genotypes J, CHC8, and BEB6 were identified in Bayi District, Gyaca, and Xaitongmoin, respectively.

MLST analysis was conducted on the *E. bieneusi*-positive samples, targeting four markers (MS1, MS3, MS4, and MS7). Among the 29 specimens, 14, 9, 11, and 15 specimens were successfully amplified at MS1, MS3, MS4, and MS7, respectively. However, ITS-positive specimens from Gyaca, Gongbo’gyamda, and Xaitongmoin were all negative in amplification of MS3 locus. At the MS4 locus, 9 of 11 sequences, successfully amplified, were obtained from Mainling and Bayi District. Altogether, sequence analysis yielded 6, 2, 2, and 3 subtypes at the MS1, MS3, MS4, and MS7 loci, respectively. Additionally, 9 samples (four from Bayi District and five from Mainling) were simultaneously positive at all four loci, forming three distinct MLGs (Table 2). The number of specimens with complete data for all four loci was limited because some specimens were not amplified at all loci, especially in the PCR analysis of the MS3 locus.

**Table 2:** Multilocus sequence typing of *E*. *bieneusi* genotypes based on minisatellite/microsatellite loci

Location	Sample ID	ITS genotype	Multilocus genotype				
			MS1	MS3	MS4	MS7	MLGs
Bayi District	6	I	–	–	–	Type1	–
	15	I	Type2	–	–	Type1	–
	29	BEB4	Type1	Type1	Type1	Type1	MLG1
	32	BEB4	Type2	Type1	Type1	Type1	MLG2
	33	BEB4	Type1	Type1	Type1	Type1	MLG1
	148	I	–	–	–	Type1	–
	306	J	Type3	Type2	Type2	Type1	MLG3
Mainling	227	BEB4	Type2	Type1	Type1	Type1	MLG2
	247	BEB4	Type2	Type1	Type1	Type1	MLG2
	250	BEB4	Type2	Type1	Type1	Type1	MLG2
	258	BEB4	Type2	Type1	Type1	Type1	MLG2
	265	BEB4	Type2	Type1	Type1	Type1	MLG2
Gongbo’gyamda	716	I	–	–	–	Type1	–
Gyaca	772	D	Type6	–	–	–	–
Xaitongmoin	1424	D	Type5	–	–	–	–
	1435	D	Type5	–	Type1	Type3	–
	1439	BEB6	Type4	–	Type1	Type2	–

## Discussion

In the present study, the overall infection rate of *Cryptosporidium* was 1.4% (8/577), which is higher than that in one report of yaks in Sichuan (1.2%, 1/84) [19]. However, molecular epidemiological data have shown the prevalence here was lower than two reports in Gansu (5.3%, 4/76 and 6.0%, 7/117), Tibet (9.1%, 4/44) and Qinghai (ranging from 3.3 to 30.0% in five studies) [10, 19–23]. Additionally, compared with dairy cattle, the distribution of *Cryptosporidium* species according to the yak age remains unknown [9]. Yaks were raised freely on pastures by herders under traditional and natural grazing condition; even herdsmen cannot make clear the age information of yaks sampled, so the precise age of individual yaks was not available during our sample collection. *C. andersoni* and *C. bovis* were identified in our study, which were identical to the species reported previously in Gansu [20]. *C. andersoni* was the dominant species in the present study, in agreement with one case in Qinghai reported by Li [22], whereas *C. parvum* was the most common species in the central-western region of China reported by Qi [19], and *C. bovis* was the dominant species in one study in Gansu reported by Qin and three reports in Qinghai [10, 20, 21, 23].

Many reports suggested that humans are frequently infected with *C. hominis* and *C. parvum*, whereas *C. andersoni* and *C. bovis* are of low zoonotic risk to humans [8]. *C. bovis* has been observed in human with diarrhea from India, Australia, and Egypt [24–26]. More studies are required to clarify the potential zoonotic transmission of *C. andersoni* and *C. bovis*. Additionally, the high-resolution genotyping tool MLST has been developed to characterize the population genetics and transmission of *C. andersoni* [27]. Over twenty MLST subtypes for *C. andersoni* have been identified in animals, most of which were isolated in dairy cattle [27–32]. Because of the limited number of isolates, four of seven *C. andersoni*-positive specimens yielded one subtype (A5,A4,A2,A1). However, the subtype (A4,A4,A4,A1) was reported to be most common in dairy and beef cattle in Henan [29], Shaanxi [31], and Heilongjiang [28]; dairy cattle in Guangdong [32]; and He cattle in Xinjiang [30], whereas the subtype (A2,A4,A2,A1) was the most prevalent subtype for dairy cattle in Xinjiang [30].

Compared with previous data obtained from other reports in yaks, the prevalence of *G*. *duodenalis* in our study (1.7%, 10/577) was slightly higher than that reported in Sichuan (1.2%, 1/36), and *G*. *duodenalis* was not detected from 44 yaks in another report from Tibet [19]. The infection rate here was lower than two reports from Gansu (1.9%, 4/208 and 3.4%, 3/117) [19, 33], and Qinghai (3.3–10.4% in four reports) [10, 19, 34, 35]. Differences in prevalence can be explained by some factors in the molecular epidemiological data, including geographic separation, immunity status, sample sizes, seasons, examination methods, ecological conditions, and management systems.

Accompanied with strong host specificity, *G*. *duodenalis* assemblage E is the dominant species in yaks, with only two reports of infection occurring with zoonotic assemblages A and B [12]. All positive products in the present study were in assemblage E, which was consistent with studies in Gansu, Sichuan, and Qinghai [19, 33]. However, two samples of assemblage A have been documented in yaks in Qinghai, and assemblage B was first detected in yaks in Qinghai [10, 34]. To date, assemblage E has been commonly identified in yaks and other domestic mammals, even though the eight assemblages are thought to have different host ranges [11]. An increasing number of documented cases of humans infected with assemblage E have been reported in Egypt, Brazil, and Australia [36–40]. Further studies are required to clarify the reservoirs and transmission routes of *G*. *duodenalis* assemblage E.

*E*. *bieneusi* has been observed in a wide variety of vertebrate hosts with worldwide distribution and importance in veterinary medicine [4]. It has high prevalence and high genetic diversity in multiple provinces and municipalities in China [41]. However, few molecular epidemiology data are available to understand the occurrence and genotypes of *E. bieneusi* in Tibet*.* We determined the prevalence and genetic diversity of *E*. *bieneusi* among yaks in five districts of Tibet, and the infection rate (5.0%, 29/577) was higher than previously reported in one study in Gansu (1.1%, 4/353) [16], but lower than that of two studies in Qinghai (7.0%, 40/554 and 7.2%, 40/554) [15, 17].

Sequence analyses indicated the presence of six distinct genotypes, of which BEB4 was predominant, followed by I, D, J, CHC8, and BEB6. Phylogenetic analysis revealed that genotype D, firstly detected in yaks, was clustered into group 1 (high zoonotic potential), and the other five genotypes belonged to group 2 (Fig. 1). The dominant genotype in our study, BEB4, was also the most common genotype in Gansu and one case in Qinghai, in which J was predominant in another report in Qinghai. Fourteen genotypes have been detected in yaks among four studies, including the present study, of which genotypes CHN11, CHN12, CHN14, WCY1, and D belong to group 1 and the remainder belong to group 2 [15–17]. Genotype D, observed in Gyaca and Xaitongmoin, has been commonly found in humans, nonhuman primates, and domestic and wild animals [42–49]. It is plausible that genotypes in group 2 have low zoonotic risk, yet genotypes J, BEB4, and BEB6 have been identified in humans in some studies [50–53]. Thus, the zoonotic potential and public health significance of genotypes in group 2 may be different than previously believed.

Based on the ITS locus, the current standard molecular marker for identification of *E*. *bieneusi*, the MLST technique has been applied to understand the transmission of *E*. *bieneusi* in humans and animals [54–56]. Because of the limited number of sequences obtained from the MS3 locus, only nine samples were simultaneously identified at all four loci (Table 2). Three distinct MLGs were formed, with two from genotype BEB4 and one from genotype J.

One study suggested that most herds in Tibet obtain drinking water from rivers, a water source shared among yaks and other grazing animals. There is a high risk of contamination of drinking water sources by the protozoa studied here [10]. Fresh fecal materials from bovines are used as cooking fuel by herders, further increasing the possibility of infection by these pathogens. The data on genetic diversity and phylogenetic relationships of these pathogens allow an assessment of their zoonotic potential and public health significance. Moreover, such studies in regions with a distinctive geographic environment and less intensive feeding can help us understand the distribution and transmission characteristics of *Cryptosporidium*, *G*. *duodenalis*, and *E*. *bieneusi*.

## Conclusion

In this study, we focused on the prevalence and genetic diversity of three intestinal pathogens from yaks in Tibet. The infection rates for *Cryptosporidium*, *G*. *duodenalis*, and *E*. *bieneusi* were lower than those in most reports conducted in yaks of Qinghai and Gansu Provinces. One subtype for *C. andersoni* (A5,A4,A2,A1) and three MLGs for *E*. *bieneusi* (two from BEB4 and one from J) were identified using MLST. Sequence and phylogenetic analyses indicated that *C. andersoni*, *C. bovis*, *G*. *duodenalis* assemblage E, and five *E*. *bieneusi* genotypes in group 2 had a low zoonotic risk, whereas five genotype D sequences showed that yaks may act as a biological disseminator or mechanical vector in the transmission of *E*. *bieneusi* to humans.

## Methods

### Study site and sample collection

**Fig. 2:**
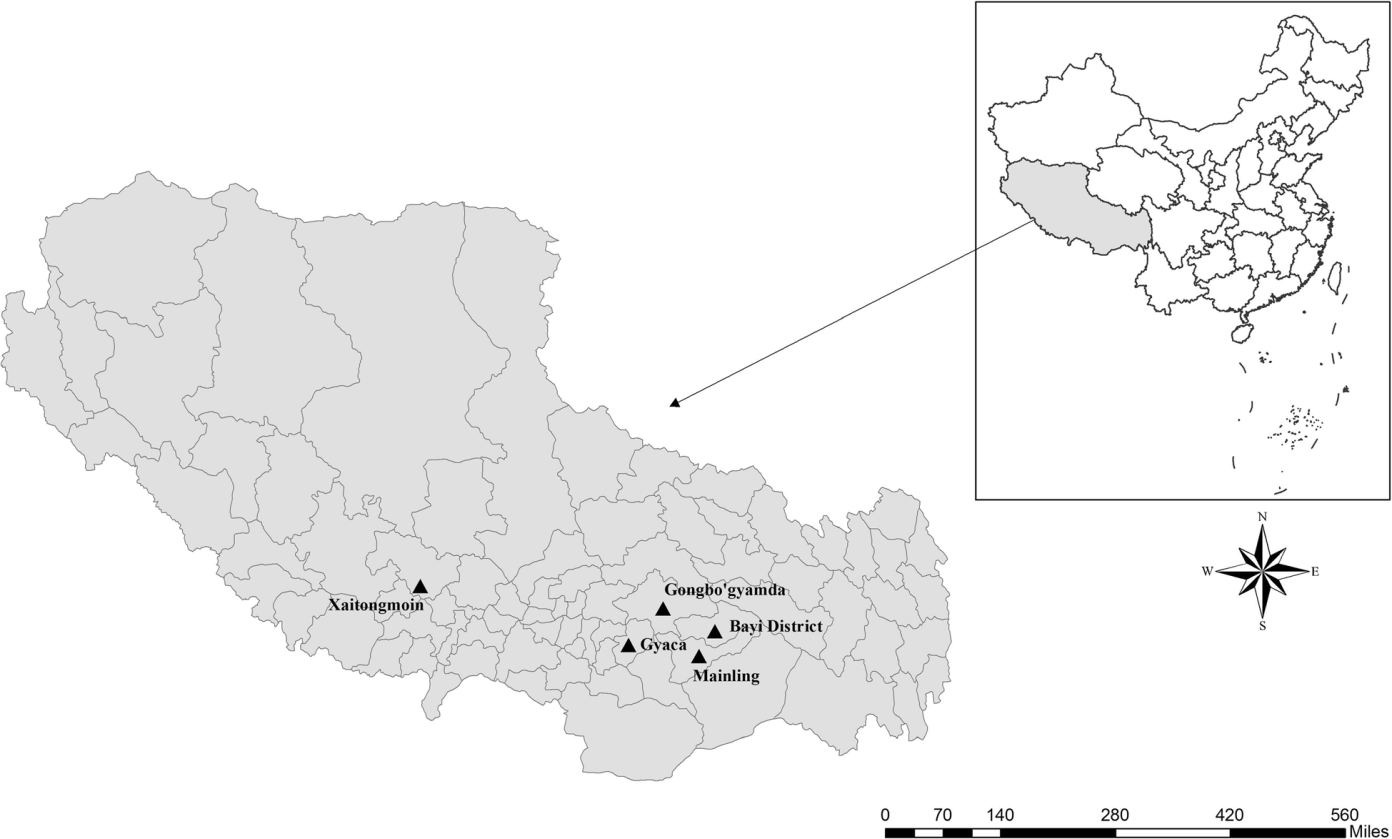
Geographic map of the sampling locations in Tibet, China. The figure was originally designed by the authors under the software ArcGIS 10.2. The original vector diagram imported in ArcGIS was adapted from National Geomatics Center of China (http://www.ngcc.cn). The map has been modified and assembled according to permission and attribution guidelines

From June 2016 to July 2016, 577 fecal specimens (approximately 30 g each) were collected on the rangelands of Mainling, Gongbo’gyamda, and Bayi District (Nyingchi), Gyaca (Lhoka), and Xaitongmoin (Shigatse) in Tibet (Fig. 2). We sought yaks defecating, and tried our best to avoid repeated sampling. Each sample was collected from the ground immediately after defecation using a sterile disposable latex glove. To avoid fecal material that had contacted the ground, care was taken to gather only the top layer of the feces. No obvious clinical signs were observed in these yaks, except for one case of diarrhoea. The fresh samples were placed in clean plastic containers marked with relevant information, transported to the laboratory, and stored in 2.5% potassium dichromate solution at 4 °C for use in subsequent DNA extraction.

### DNA extraction and PCR amplification

To reduce the effect of potassium dichromate, each specimen was washed three times with distilled water before centrifugation at 1500×*g* for 10 min at room temperature followed by DNA extraction. According to the procedure recommended by the manufacturer, genomic DNA was extracted from 200 mg of each specimen using the E.Z.N.A.R® Stool DNA (Omega Biotek Inc., Norcross, GA, USA), and the obtained DNA was stored at − 20 °C until used in the PCR analysis.

To screen for *Cryptosporidium* spp., *G*. *duodenalis*, and *E*. *bieneusi* infection, the corresponding loci were used as described previously. *Cryptosporidium* was identified by the SSU rRNA gene [57]; *G. duodenalis* was identified based on the SSU rRNA [58] and *tpi* genes [59]; and *E. bieneusi* was detected by nested PCR targeting the partial region of ITS [60] (Table 3). With the Applied Biosystems 2720 Thermal Cycler (Applied Biosystems, Foster City, USA), the amplification for *Cryptosporidium* and *E. bieneusi* was performed in 25 μL volume with 1 μL extracted DNA sample, 2.5 μL 10 × KOD-Plus PCR buffer, 2.5 μL dNTPs (2 mM), 1.5 μL MgSO4 (25 mM), 0.5 μL each primer (25 μM), 16 μL double distilled water, and 0.5 μL KOD-Plus amplification enzyme (1 unit/μL) (ToYoBo Co., Ltd., Osaka, Japan). The PCR reaction mixture reactions for *G*. *duodenalis* loci were conducted: 2.5 μL 1 × PCR buffer, 2 μL dNTPs (1.25 mM), 0.3 μL each primer (25 μM), 0.2 μL rTaq DNA polymerase (1 unit/μL) (*Takara* Shuzo Co., Ltd), 2 μL of DNA sample, 16.7 μL double distilled water, and 1 μL of bovine serum albumin (10 mg/mL). Samples positive for *C. andersoni* and *E*. *bieneusi* were selected and characterized by the multilocus sequence typing (MLST) technique, using nested PCR amplifications targeting the microsatellite/minisatellite loci (MS1, MS2, MS3, and MS16 for *C. andersoni*), and (MS1, MS3, MS4, and MS7 for *E*. *bieneusi*) [27, 54]. Samples that were positive at all four loci were used to deduce the MLGs of *C. andersoni* and *E*. *bieneusi*. The pre-existing positive DNA samples stored in lab were set for positive control. Controls were included in all PCRs runs.

**Table 3:** Primer sequences and reaction conditions used in nested PCR amplifications

Locus	Primer sequences (5′-3′)	Nucleotide fragment (bp)	Annealing temperature (°C)	Reference
*Cryptosporidium* SSU rRNA	SSU-F2: TTCTAGAGCTAATACATGCG	840	55	[57]
	SSU-R2:CCCATTTCCTTCGAAACAGGA			
	SSU-F3:GGAAGGGTTGTATTTATTAGATAAAG		55	
	SSU-R4:CTCATAAGG TGCTGAAGGAGTA			
*G*. *duodenali* SSU rRNA	Gia2029: AAGTGTGGTGCAGACGGACTC	292	55	[58]
	Gia2150c: CTGCTGCCGTCCTTGGATGT			
	RH11: CATCCGGTCGATCCTGCC		59	
	RH4: AGTCGAACCCTGATTCTCCGCCCAGG			
*G*. *duodenalis tpi*	AL3543: AAATIATGCCTGCTCGTCG	530	50	[59]
	AL3546: CAAACCTTITCCGCAAACC			
	AL3544: CCCTTCATCGGIGGTAACTT		50	
	AL3545: GTGGCCACCACICCCGTGCC			
*E. bieneusi* ITS	EBITS3: GGTCATAGGGATGAAGAG	389	57	[60]
	EBITS4: TTCGAGTTCTTTCGCGCTC			
	EBITS1: GCTCTGAATATCTATGGCT		55	
	EBITS2.4: ATCGCCGACGGATCCAAGTG			

The secondary PCR products were separated by 1% agarose gel electrophoresis following staining with DNA Green (TIANDZ, Beijing, China) and visualized on a UV transilluminator. The positive secondary amplification products were sequenced on an ABI PRISM™ 3730 XL DNA Analyzer using the BigDye Terminator v3.1 Cycle Sequencing Kit (Applied Biosystems, Foster City, CA, USA). Data accuracy was confirmed with two-directional sequencing.

### Data analysis

To characterize the phylogenetic analysis of sequences of the ITS regions obtained here and reference sequences downloaded from GenBank. Bayesian inference (BI) and Monte Carlo Markov Chain (MCMC) methods were used to construct phylogenetic trees in MrBayes, version 3.2.6 (http://mrbayes.sourceforge.net/). Determined by ModelTest, version 3.7 (http://www.molecularevolution.org/), the general time reversible model (GTR + G) was the best-fit nucleotide substitution model. The number of substitutions (Nst) was set at six, and posterior probability values were calculated by running 1,000,000 generations with four simultaneous tree-building chains. Trees were saved every 1000th generation. At the end of each run, the standard deviation of the split frequencies was < 0.01, and the potential scale reduction factor approached one. A 50% majority rule consensus tree was constructed for each analysis using the final 75% of the trees generated via BI. FigTree, version 1.3.1 (http://tree.bio.ed.ac.uk/software/figtree/), was used to visualize and edit the maximum clade credibility by these analyses.

### Statistical analysis

The infection rates of 95% confidence intervals (CI) were calculated by Wald method of SPSS 22.0 version (SPSS Inc., Chicago, IL, United States). Differences in corresponding infection rates among locations were examined by the Chi-square test, and differences were considered significant at *P* < 0.05.

## Data Availability

All data generated and/or analyzed during this study are included in this published manuscript. The 24 representative nucleotide sequences obtained have been deposited in GenBank database under the following accession numbers: MK139948 for *Cryptosporidium*, MK140454 to MK140456 and MK165410 for *C. andersoni* MLST subtype, MK139942 to MK139947 for *E*. *bieneusi*, MK140446 to MK140453 and MK165405 to MK165409 for *E*. *bieneusi* MLST subtype.

## Abbreviations

CAFO: Concentrated Animal Feeding Operation
CI: Confidence intervals
ITS: Internal transcribed spacer
MLST: Multilocus sequence typing
SSU rRNA: Small subunit rRNA
*tpi*: triosephosphate isomerase
